# Long non-coding RNAs in ischemic stroke

**DOI:** 10.1038/s41419-018-0282-x

**Published:** 2018-02-15

**Authors:** Mei-Hua Bao, Vivian Szeto, Burton B. Yang, Shu-zhen Zhu, Hong-Shuo Sun, Zhong-Ping Feng

**Affiliations:** 1 Department of Anatomy, Histology and Embryology, Institute of Neuroscience, Changsha Medical University, Changsha, 410219 China; 2 Department of Physiology, Faculty of Medicine, University of Toronto, Toronto, ON Canada; 3 Department of Surgery, Faculty of Medicine, University of Toronto, Toronto, ON Canada; 4 Sunnybrook Research Institute and Department of Laboratory Medicine and Pathology, Faculty of Medicine, University of Toronto, Toronto, Canada

## Abstract

Stroke is one of the leading causes of mortality and disability worldwide. Uncovering the cellular and molecular pathophysiological processes in stroke have been a top priority. Long non-coding (lnc) RNAs play critical roles in different kinds of diseases. In recent years, a bulk of aberrantly expressed lncRNAs have been screened out in ischemic stroke patients or ischemia insulted animals using new technologies such as RNA-seq, deep sequencing, and microarrays. Nine specific lncRNAs, antisense non-coding RNA in the INK4 locus (ANRIL), metastasis-associate lung adenocarcinoma transcript 1 (MALAT1), N1LR, maternally expressed gene 3 (MEG3), H19, CaMK2D-associated transcript 1 (C2dat1), Fos downstream transcript (FosDT), small nucleolar RNA host gene 14 (SNHG14), and taurine-upregulated gene 1 (TUG1), were found increased in cerebral ischemic animals and/or oxygen-glucose deprived (OGD) cells. These lncRNAs were suggested to promote cell apoptosis, angiogenesis, inflammation, and cell death. Our Gene Ontology (GO) enrichment analysis predicted that MEG3, H19, and MALAT1 might also be related to functions such as neurogenesis, angiogenesis, and inflammation through mechanisms of gene regulation (DNA transcription, RNA folding, methylation, and gene imprinting). This knowledge may provide a better understanding of the functions and mechanisms of lncRNAs in ischemic stroke. Further elucidating the functions and mechanisms of these lncRNAs in biological systems under normal and pathological conditions may lead to opportunities for identifying biomarkers and novel therapeutic targets of ischemic stroke.

## Facts

- The expression levels and types of a large number of lncRNAs are susceptible to change during and/or following ischemic stroke insults.
- Nine lncRNAs, including ANRIL, MALAT1, N1LR, MEG3, H19, C2dat1, FosDT, SNHG14, and TUG1, are involved in cell death, angiogenesis, and inflammation during ischemic stroke.
- MEG3, H19, and MALAT1 might be related to neurogenesis in ischemic stroke.

## Open questions

- How do lncRNAs regulate gene expression?
- What lncRNAs are involved in ischemic stroke?
- What are the functions and underlying mechanisms of the lncRNAs in ischemic stroke?

## Ischemic stroke

Stroke is the second leading cause of disability and mortality above the age of 60. Every year, 15 million people worldwide suffer from stroke. Ischemic stroke accounts for about 87% of all strokes. For the treatment of ischemic stroke, tissue plasminogen activator (or Alteplase IV r-tPA) is the only FDA-approved drug. However, tPA has a very narrow therapeutic window of 3 h from the onset of a stroke or up to 4.5 h in certain eligible patients. This shortcoming of plasminogen activator therapy urges researches seeking for new strategies, especially therapies concerning neuroprotection, neurogenesis, and angiogenesis. A better understanding of the cellular and molecular mechanisms underlying the pathological process of stroke and post-stroke recovery may provide new methods complementary to r-tPA for ischemic stroke therapy^1–3^.

## Long non-coding RNA (lncRNA)

At least 98% of the human genome has non-protein-coding regions. Among the 98% regions, 80% are transcribed to RNAs. The transcripts of these regions have been regarded as transcriptional “noise” for a long time. Recently, the expression, function, and mechanisms of these non-coding RNAs have drawn wide attention. According to the length, non-coding RNAs are divided into small (<200 nt) RNAs (microRNAs and transfer RNAs) and long (>200 nt) RNAs (ribosomal RNAs and lncRNAs)^4,5^.

### Discovery of lncRNA

**Fig. 1:**
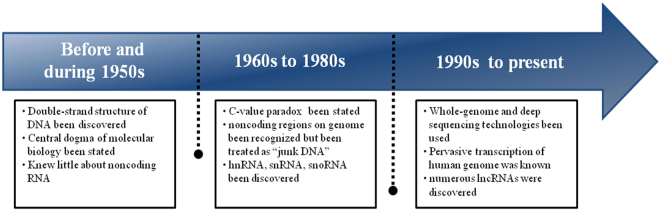
The timeline of lncRNA discovery

The discovery of lncRNA can be divided into three periods: (1) before and during the 1950s, (2) 1960s to 1980s, and (3) 1990s to present (Fig. 1).

During the 1950s, double-strand DNA structure was discovered by James Watson and Francis Crick. The Central Dogma of molecular biology stating the flow of genetic information was later described by Francis Crick in 1958^6^. Since then, scientists noticed that the amount of DNA had little correlation with an organism’s size or complexity^7,8^. For example, animals with relatively simpler and more primitive characteristics like the salamander have a genome 15 times larger than that of the humans^9^. This is regarded as the C-value paradox^9^. The paradox was reasoned by the discovery of those parts of the genome which may not encode protein. In salamander, a large portion of the DNA may not involve in protein coding or regulatory functions. Thus, those non-protein-coding RNAs were treated as “junk DNA”^8,10^.

In the 1970s and 80s, some hypothesized that this “junk DNA could aid in genome integrity, gene regulation, and mRNA procession”^8,11–14^. Increasingly, different kinds of non-coding RNAs such as “heterogeneous nuclear RNAs” (hnRNAs)^15^, small nuclear RNAs (snRNAs), and small nucleolar RNAs (snoRNAs)^16,17^ were discovered, suggesting that “junk DNA” might not be as simple.

Whole-genome analysis technologies developed in the late 1990s and early 2000s allowed a more comprehensive understanding of genome transcription. It was estimated that 70–90% of the human genome was transcribed to RNA, and over 68% of human transcriptome is lncRNA^18–20^. Some of the lncRNAs and their characteristics were reported in the early 1990s, for example, H19^21^ and *Xist*^22,23^. With the help of deep sequencing, numerous lncRNAs have been identified^24^. Although the idea of “transcriptional noise” still resonated in this field, some non-coding RNAs, including microRNAs, lncRNAs, and circular RNAs were discovered, classified, with some functions identified^25–30^.

### Mechanisms of lncRNA functions

**Fig. 2:**
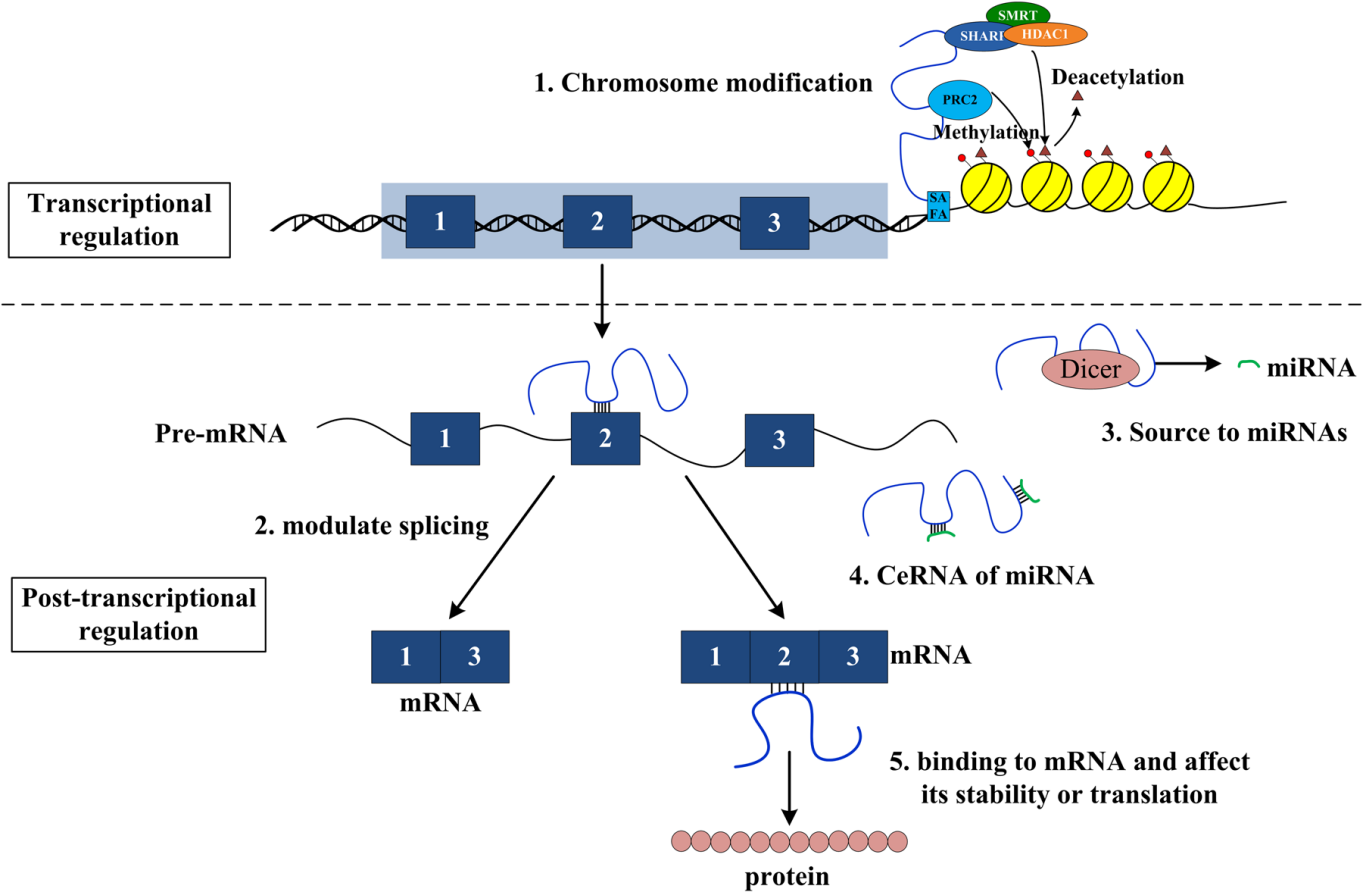
Mechanisms of lncRNA functions. 1. Chromosome modification. The lncRNA Xist scaffolds and recruits different kinds of regulatory proteins, such as SMRT/HDAC1-associated repressor protein (SHARP), binds to chromatin by scaffold attachment factor A (SAFA), and promotes histone deacetylation on X chromosomes. Xist also recruits PRCs and triggers methylation of lysine H3K9 and H3k27. 2. Modulating splicing. lncRNA binds to pre-mRNA and blocks the binding of spliceosome to target sequence, results in the formation of splicing variants. 3. Source of miRNA. Many lncRNA genes contain embedded miRNA sequence in its introns or exons, which harbors miRNAs. 4. CeRNA to miRNAs. Some lncRNAs contain complementary binding sites to certain miRNA, which soak up the target miRNA and result in the reduction of miRNA functions in cells. 5. Binding to mRNA and affect its stability or translation

The mechanisms for the actions of lncRNAs include both transcriptional and post-transcriptional regulation. In the transcriptional level, lncRNAs regulate and modify chromosomes, leading to the alteration of gene expression. In the post-transcriptional level, lncRNAs work as competing endogenous RNA (ceRNA) and miRNA source, and are involved in RNA degradation (Fig. 2).

#### The transcriptional regulation

Many lncRNAs localize in the nucleus, and studies have demonstrated their effects on gene expression^31,32^. In the nucleus, the lncRNAs scaffold and recruit different kinds of chromatin regulatory proteins, identify and interact with chromatin in specific sites by methods of three-dimensional (3D) proximity or affinity, integrate and orchestrate the shape of the chromosome, and then suppress or activate the expression of genes, or change the modification (acetylation or methylation) of the chromatin.

For instance, Xist (X inactive specific transcript) is involved in X chromosome inactivation (XCI)^33,34^. Xist is expressed only on inactive X chromosomes (Xi), not on active X chromosomes (Xa)^35^. During the initiation of XCI, the expression of Xist recruits SMRT/HDAC1-associated repressor protein (SHARP), binds to chromatin by scaffold attachment factor A (SAFA), and promotes histone deacetylation on X chromosomes by histone deacetylase 3 (HDAC3). The deacetylation accompanied by demethylation of H3K4 ejects the RNA polymerase II, leading to the inactivation of X chromosomes^36–38^. Xist also recruits other protein complexes such as polycomb repressive complex 1 (PRC1) and PRC2^39^. The PRCs then trigger the methylation of lysine H3K9 and H3k27 on histones^40^.

#### The post-transcriptional regulation

After transcription, lncRNAs regulate the gene expression either directly by affecting the RNA splicing and RNA degradation, or indirectly through regulating miRNA functions.

RNA splicing is a crucial step for a precursor messenger RNA (pre-mRNA) being transcripted into mRNA. The main method of splicing is by removing the introns of pre-mRNA and ligating the exons through spliceosome. The binding blockage of spliceosome to pre-mRNA target sequences affects the maturation of mRNAs and results in the formation of splicing variants. Some lncRNAs have sequences base-paired to the pre-mRNAs and block the splicing of these pre-mRNAs. For example, an antisense lncRNA, NAT, binds to an intron of *Zeb2* gene located at the 5ʹ-UTR. The binding of NAT to *Zeb2* pre-mRNA prevents the splicing of this intron. Since this intron contains an internal ribosome entry site (IRES) necessary for the expression of *Zeb2*, the maintenance of this intron results in an activation of its expression^41^.

LncRNAs can directly bind to mRNA and regulate the degradation of mRNA. A transcriptome-wide analysis found 18,871,097 lncRNA–RNA base-pairings in humans. These interactions could be involved in processing, stability control, and functions of 57,303 transcripts^42^. For example, antisense of beta-secretase-1 (BACE1-AS) base-pairs BACE1, stabilizes the BACE1 mRNA, and promotes the generation of amyloid-beta 1-42, which eventually aggravates the Alzheimer’s disease^43^.

LncRNAs can influence the formation or function of miRNAs to regulate gene expression. Many lncRNA genes contain embedded miRNA sequences in their introns or exons, which harbors miRNAs. Many lncRNAs act as the origination for miRNAs. Ma et al. identified 172,713 DCL1-dependent small RNAs in *Arabidopsis*^44^. Among them, 65,006 small RNAs found their loci on 5891 lncRNAs. And all the sRNAs were mapped to the registered pre-miRNAs of Arabidopsis in miRBase (www.mirbase.org/)^44^. One of the first discovered lncRNAs, H19, is an imprinted non-coding RNA which is a precursor for miR-675^45^. As miRNAs host genes, lncRNAs control the formation of the miRNAs. Moreover, lncRNAs can act as ceRNA to reduce the concentrations of miRNAs. Some lncRNAs contain complementary binding sites to certain miRNAs, which soak up the target miRNAs and result in the reduction of miRNA functions in cells^46^. In this way, the lncRNAs negatively regulate the functions of miRNAs. A bulk of lncRNAs are found in recent years that act as miRNA sponge.

## LncRNA in ischemic stroke

**Fig. 3:**
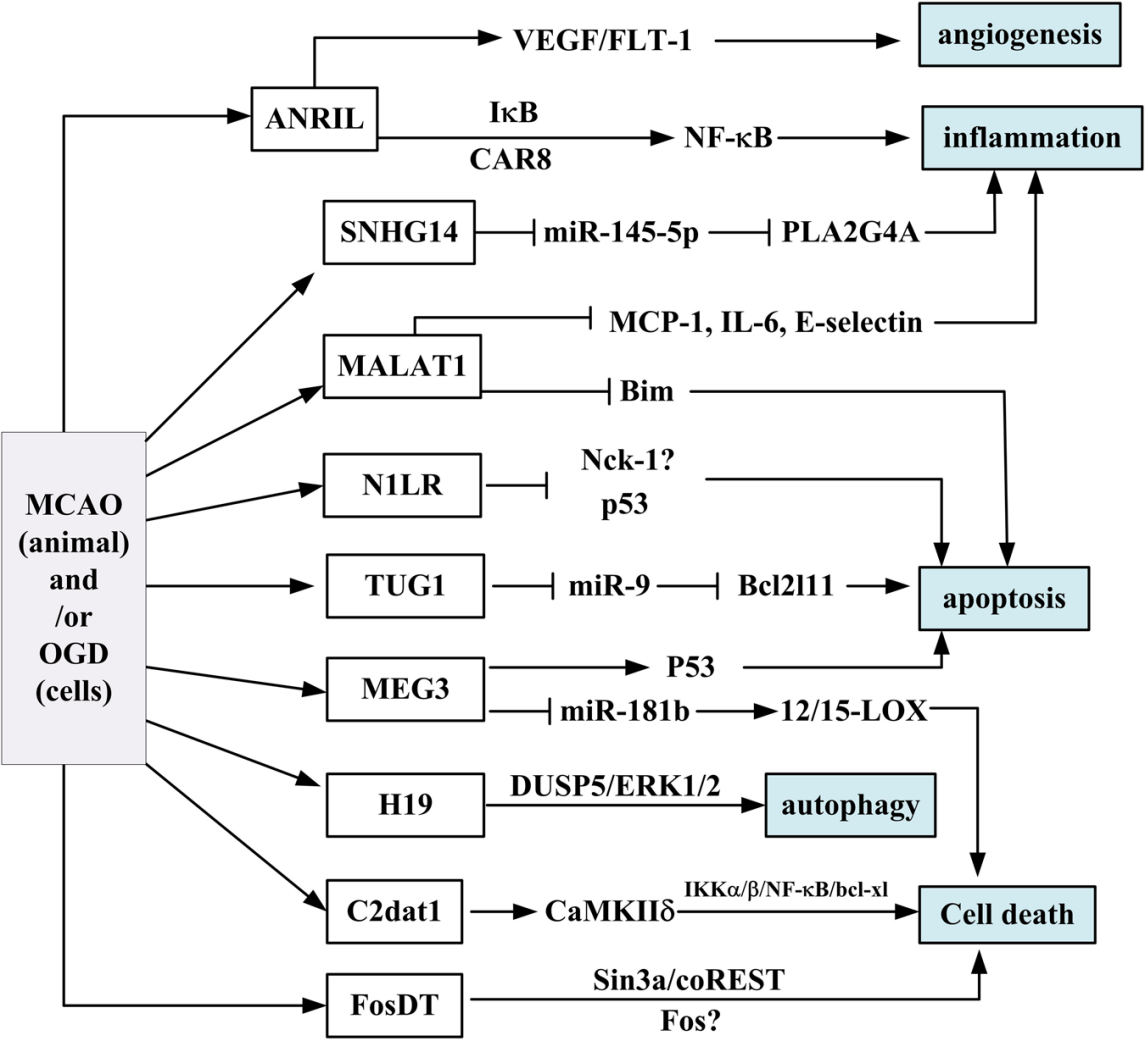
Function and signal of some ischemic stroke-related lncRNAs. MCAO middle cerebral artery occlusion, OGD oxygen glucose deprivation, ANRIL antisense non-coding RNA in the INK4 locus, MALAT1 metastasis-associate lung adenocarcinoma transcript 1, TUG1 taurine-upregulated gene 1, MEG3 maternally expressed gene 3, C2dat1 CaMK2D-associated transcript 1, FosDT Fos downstream transcript, SNHG14 small nucleolar RNA host gene 14.

The roles of some lncRNAs in ischemic stroke were elucidated recently. Hundreds of aberrantly expressed lncRNAs were identified using techniques such as microarray or RNA-seq in ischemic stroke patients or ischemic insulted animal models^47,48^. Specifically, antisense non-coding RNA in the INK4 locus (ANRIL), MALAT1, N1LR, maternally expressed gene 3 (MEG3), H19, CaMK2D-associated transcript 1 (C2dat1), Fos downstream transcript (FosDT), small nucleolar RNA host gene 14 (SNHG14), and taurine-upregulated gene 1 (TUG1) were found to affect cell apoptosis, inflammation, cell death, and angiogenesis during ischemic stroke (Table 1 and Fig. 3).

**Table 1:** The expression, function, and mechanism of some lncRNAs in ischemic stroke

**LncRNA**	**Animal or/and cells**	**Stroke model**	**Change in expression**	**Target/mechanisms**	**Function**
ANRIL	Adult male Wistar rats HUVECs	MCAO	Increased	Promote CARD8 level; promote angiogenesis and inflammation through VEGF and NF-κB pathway	Promote infarction, angiogenesis
C2dat1	C57BL/6 mice N2a cells	MCAO	Increased	Increase the expression of CaMKIIδ, and activate subsequent NF-κBsignal	Promote ischemic brain injury
		OGD/Reperfusion			
H19	SD rats SH-SY5Y cells	MCAO	Increased	Unknown	Promote cell death and autophagy.
		OGD/Reperfusion			
MALAT1	C57BL/6 mice BMEC	MCAO	Increased	Bind to Bim and E-selectin directly	Inhibit cell death and apoptosis and inflammatory factors
		OGD			
N1LR	SD rats N2a cells	MCAO/Reperfusion	Increase first and decrease gradually	Unknown	enhanced proliferation, inhibited apoptosis
		OGD/Reperfusion			
MEG3	C57 mice brain cortex N2a cell, HT22 cells	MCAO	Increased	Bind directly to p53 protein at position of DBD_270–281_; competing endogenous RNA for miR-18b to regulate 12/15-LOX expression	Promote cell death, apoptosis and infarction
		OGD	Increased		
FosDT	SHR rats	MCAO	Increased	Bind to Sin3a and coREST, and influence the subsequent genes of REST.	Promote ischemic brain damage in rats
SNHG14	C57BL/6 mice Bv-2 cells	MCAO	Increased	Increased the expression of PLA2G4A by inhibition of miR-145-5p	Promotion of infarction and apoptosis
		OGD	Increased		
TUG1	Rat SH-SY5Y cells	MCAO	Increased	Decrease the miRNA-9 expression and increase Bcl2l11 protein	Promote cell apoptosis
		OGD	Increased

### ANRIL

ANRIL is an antisense non-coding RNA co-clustered with p15/CDKN2B-p16/CDKN2A-p14/ARF locus in the position of chromosome 9p21, first identified by Pasmant in 2007^49^. It is presented in more than eight splice variants at the length of ~3.9 kb^50^. Chromosome 9p21 is a risk locus for cardiovascular diseases and some carcinomas^51,52^. In cancers, ANRIL can be activated by hypoxia-inducible factor-1α and c-Myc^53,54^. ANRIL regulates gene expression by binding to PRC1 or PRC2 and mediates gene silencing at the INK4b-ARF-INK4a locus^55^. Increased expression or mutations in ANRIL are also associated with atherosclerosis, coronary artery disease, and stroke^56–59^. ANRIL expression correlated with chromosome 9p21.3 variants was thought to be a novel genetic marker for the risk of stroke and its recurrence^60^.

In cerebral infarction rat models, the expression of ANRIL in the cortex infarction was significantly increased (more than 1.5 folds of normal control)^61^. Increased ANRIL then activated the vascular endothelial growth factor (VEGF)/VEGF receptor 1 (FLT-1) and IκB/NF-κB pathways^61^, subsequently promoting angiogenesis and inflammation processes. VEGF is a strong stimulator for angiogenesis. The binding of VEGF to its receptor, FLT-1, is essential during embryological vasculogenesis and persists in adult animals to maintain the function of endothelium^62^. NF-κB is a multifunctional transcription factor. The binding of NF-κB with IκB sequesters the complex in the cytoplasm. Under certain stresses, such as oxidative stress, cytokines, ultraviolet irradiation, and bacterial or viral antigens, NF-κB dissociates from IκB, then translocates to the nucleus and regulates the expression of genes responsible for both innate and adaptive immune response^63^. Through the activation of VEGF/FLT-1 pathway and IκB/NF-κB pathway, ANRIL may play a role in pro-angiogenesis and pro-inflammation.

Caspase recruitment domain family member (CARD) 8 is another target of ANRIL. CARD8 gene encodes for CARD8 protein also known as TUCAN/CARDINAL, which is an inhibitor of NF-κB pathway^64^. Studies have shown that an SNP in CARD8 (rs2043211), which changes A to T and reduces the expression of CARD8, was associated with decreased risk of ischemic stroke^65^. The increase or decrease of ANRIL in HepG2 cells promoted or inhibited the expression of CARD8, respectively^66^. Activation of ANRIL inhibits NF-κB through the activation of CARD8, and thus may inhibit the process of inflammation.

Taken together, increased ANRIL in ischemic stroke promotes angiogenesis through VEGF/FLT-1 pathway and regulates inflammation by both promotion and inhibition of the NF-κB pathway.

### MALAT1

MALAT1 is among the first lncRNAs identified as promoting metastasis and proliferation of different cancers through alternative splicing and gene expression^67–71^. MALAT1 is a well conserved, stable, and abundant lncRNA (~7 kb), existing in different species^72^. Recently, MALAT1 expression was found in vascular endothelial cells, skeletal muscle, cardiomyocytes, and was suggested to participate in the pathological myogenesis and angiogenesis^73–76^. MALAT1 is abundantly expressed in cell nucleus speckles, a domain that is related to the pre-mRNA procession, and might be involved in the organization or regulation of gene expression^77^. MALAT1 also affects pre-mRNA splicing through interaction with phosphorylated splicing factors of precursor messenger RNAs (SR) proteins^78^. In cancers, the expression of MALAT1 is upregulated by hypoxia or HIF-1α^79,80^.

The functions of MALAT1 in ischemic stroke were identified recently. In both oxygen glucose deprivation (OGD) endothelial cells and middle cerebral artery occlusion (MCAO) mouse models of stroke, MALAT1 was increased significantly (6.05-fold higher than that of the control group in OGD endothelial cells)^48^. MALAT1 knock-out presented with larger brain infarct size, worse neurological scores, and reduced sensorimotor functions post-MCAO^81^. Genetic ablation of MALAT1 in mice reduced the vascular growth in retinas^73^, consistent with other studies on cancer angiogenesis^73,82,83^. Since cerebral vasculature is important in improving clinical outcomes in the post-ischemic recovery phase, increasing MALAT1 in ischemic stroke suggested a protective and healing property of the ischemic brain.

Silencing of MALAT1 led to the increase in pro-apoptotic factor Bim and expression of pro-inflammatory cytokines monocyte chemotactic protein-1 (MCP-1), interleukin 6 (IL-6), and E-selectin in brain microvascular endothelial cells (BMECs), as well as in ischemia insulted mice brain^81^. These results indicate that the protective effect of MALAT1 on cerebral ischemic insults occured through inhibiting endothelial cell death and inflammation.

### MEG3

MEG3 is an ~1.6 kb imprinted gene located on the chromosome 14q32.3 DLK1 locus, and expressed in many normal tissues in human. The MEG3 acts as an anti-proliferative gene in cancer and is treated as a cancer suppressor^84^. The loss of MEG3 expression caused the formation of various types of cancers, while over-expression of MEG3 inhibited them^85,86^. Many mechanisms, such as gene deletion, hypermethylation of the intergenic differentially methylated region, and promoter hypermethylation, contribute to the loss of MEG3 expression in tumors^84^.

Recently, the function and expression of MEG3 in the neural system and ischemic stroke were discovered^87,88^. MEG3 presents as a cytotoxic factor for ischemic injury in both MCAO mice and OGD neurons. In both MCAO mice brain and OGD-treated neuronal HT22 cells, the expression of MEG3 increased significantly (more than 3 fold of control in both MCAO mouse and OGD-treated HT22 cells)^88^. Inhibition of MEG3 by MEG3 siRNAs decreased the infarction and edema volume and increased the neurobehavior score in MCAO mice^88^. Meanwhile, the increase in MEG3 was accompanied by the increase in neuron death and apoptosis. Further studies found that p53 and 12/15-Lipoxygenase (12/15-LOX) were involved in the functions of MEG3. P53 plays crucial roles in DNA repair: it arrests the cell cycle in G1/S phase to facilitate DNA repair and initiates apoptosis when DNA damage proves to be irreparable^89^. Therefore, p53 is treated as a cancer suppressor and thought to be essential for cellular and genetic stability. In cerebral ischemia insulted mice, an increase in MEG3 was found to promote the expression of p53 by binding directly to the DBD_270–281_ site of p53 gene and to facilitate the neuron apoptosis^88^. The dissociation of p53 from MEG3 suppressed neuron apoptosis and reduced infarction volume in MCAO mice, indicating that MEG3 functions through p53^88^. 12/15-LOX is a main isoform of lipoxygenases, a group of enzymes that catalyze the formation of hydroperoxides from polyunsaturated fatty acids such as linoleic acid and arachidonic acid. Studies show that neuronal 12/15-LOX was robustly activated in the injured brain. It mediated oxidative stress-induced neuronal dysfunction contributing to neuronal death after cerebral ischemia^90,91^. MiR-181b is a key regulator of 12/15-LOX expression; the over-expression of miR-181b inhibited the production of 12/15-LOX-1^87^. LncRNA-MEG3 acts as a competitive endogenous RNA in the miR181b-12/15-LOX cascade. The upregulation of MEG3 in MCAO mice or OGD neurons acted as microRNA sponge and competitively inhibited the effects of miR-181b on 12/15-LOX, leading to upregulation of 12/15-LOX and subsequent neuronal death^87^.

### LncRNA-H19

LncRNA-H19 is a 2.3 kb RNA coded by H19 gene^92^. It is a highly conserved imprinted gene which is expressed only in maternal allele. H19 was firstly found to play important roles in the embryonal development and growth control^93^. Both maternal and paternal H19 alleles are expressed at first stage of embryonal development (6–8 weeks gestation). However, only maternal chromosomes express H19 after 10 weeks of gestation^94^. H19 gains its function in controlling embryo growth through targeting another imprinted gene, Igf2^95^. The hypermethylation in the promoter of H19 gene and allele-specific methylation of 3′ portion of H19 may be related to the change in the expression of H19^94^.

However, in some pathological conditions, such as cancer and oxidative stress, H19 expression is re-evoked^96–98^. For instance, circulating H19 levels significantly increased in stroke patients compared with healthy controls^99^. The plasma level of H19 was suggested to have high diagnostic value for ischemic stroke^99^. The expression of H19 was upregulated both in MCAO/reperfusion rat brain and OGD/reoxygenation SH-SY5Y cells^100^. A variation in H19, rs217727, was associated with a higher risk of ischemic stroke^100^. Further studies found that inhibition of H19 protected SH-SY5Y cells from OGD/R-induced cell death and autophagy significantly. Dual specificity phosphatase 5 (DUSP5)-ERK1/2 axis was shown to participate in the pro-autophagy effects of H19. DUSP5 is a mitogen-activated protein kinase phosphatase, which inhibits the ERK1/2 pathway and suppresses autophagy^100–102^. The increase in H19 levels inhibited DUSP5, thus activating ERK1/2 and autophagy. Excessive autophagy during cerebral ischemic reperfusion injury induces apoptosis, necrosis, and autophagic death of neurons^103^.

### TUG1

TUG1 is a 7.2 kb lncRNA, which was initially detected in taurine-treated mouse retinal cells. The upregulation of TUG1 is essential for retinal development^104^. Recently, TUG1 was found to be related to the progression of human diseases. In many cancers, such as osteosarcoma, glioma, and gastric cancer, TUG1 levels increased dramatically and promoted the metastasis^105^. The functional studies found that TUG1 binds to the PRC2, and regulates the methylation of H3K27me3 to repress gene expression^106,107^. TUG1 also acted as a gene sponge for microRNAs, including miR-9, miR-26a, miR-144, miR-300, MIR-377, miR-299, and miR-335^108–113^.

In the neural system, TUG1 was upregulated in the brain of MCAO rats and OGD-treated SH-SY5Y cells (1.51–2.79 folds of control)^109^. The upregulation of TUG1 resulted in a larger infarction volume in ischemic insulted rats and a higher apoptosis rate in OGD-treated SH-SY5Y cells. Further studies found that TUG1 interacted with miR-9 and sequestered it directly^109^. MiR-9 is a microRNA highly expressed in neurogenic regions^114^. It inhibits bcl2l11, a pro-apoptosis protein in ischemic injury^109^. Inhibition of miR-9 by TUG1 led to a decline of miR-9 and subsequently weakened the inhibition effects of miR-9 on Bcl2l11, leading to a cytotoxic effect.

### N1LR

LncRNA-N1LR is a 1.8 kb lncRNA located on chromosome 9 and overlaps sequence with 5ʹ-UTR of Nck1 in mice. To date, only one study was conducted to look into the function of N1LR^115,116^. N1LR is a lncRNA originally found aberrantly expressed in cerebral ischemia/reperfusion rat model in 2016^116^. 0.5 h of MCAO followed by 24 h reperfusion increased the production of N1LR (more than 3 folds of control), while a longer time of MCAO (1–2 h) followed by 24 h reperfusion inhibited the production of N1LR significantly (about 50% of control). With the decrease of N1LR, infarct volume was increased dramatically. Decrease in N1LR expression and increase in injury (cell apoptosis) were also found in OGD/R-treated N2a cells. Over-expression of N1LR reduced the apoptosis induced by OGD/R in N2a cells through prevention of the activation of p53^116^. These results indicate that N1LR may be necessary for the neurons to resist ischemic injury. Interestingly, genome location analysis and RACE assay show that N1LR overlaps with the 5ʹ-UTR of protein-coding gene *Nck1*. *Nck1* is thought to be involved in cellular remodeling, glucose tolerance, and insulin signaling. *Nck1* is increased in ischemia insulted rat brain. Knockdown of lncRNA-N1LR also resulted in a modest increase of *Nck1* expression. However, over-expression of lncRNA-N1LR had no obvious effects on *Nck1* expression^115,116^. Therefore, whether and how lncRNA-N1LR interacts with *Nck1* in ischemic stroke is still unclear.

### C2dat1

C2dat1, a CaMK2D-associated lncRNA, was first discovered in a lncRNA array analysis of MCAO insulted rat brain in 2016^117^. In a later study, a higher expression level of C2dat1 was found in osteosarcoma cells, where it promotes cell viability, migration, and invasion through interaction with miR-34-5p, and Sirt1^118^. C2dat1 is a sense lncRNA which overlaps with intron 13–15 and exon 14 of CaMK2D gene in the genome. In both MCAO insulted mouse brain or OGD/R-treated N2a cells, the expression of C2dat1 was increased significantly (more than 4 folds after 12 h reperfusion). This increase in C2dat1 was accompanied by an increase in CaMK2D^117^. Moreover, C2dat1 was mainly located in the nucleus of N2a cells, and the inhibition of C2dat1 using si-C2dat1 led to a suppression of CaMK2D mRNA and protein. These results indicate that C2dat1 may interact with CaMK2D directly. However, the precise regulation pattern between C2dat1 and CaMK2D still needs further investigation.

Functionally, inhibition of C2dat1 or CaMK2D by C2dat1 siRNAor CaMK2D siRNA promoted OGD/R-induced N2a cell death, indicating a neuroprotective effect of C2dat1 and CaMK2D^117^. CaMK2D is highly expressed in brain and muscle tissues and mediates the intracellular Ca^2+^ signals^119,120^. Activation of CaMK2D induced both spontaneous and β-adrenergic stimulated arrhythmias^121^, and aggravates cardiomyocyte hypertrophy^122^. In OGD/R-treated N2a cells, activation of CaMK2D induced phosphorylation of IKKα/β, degradation of IκB, activation of NF-κB, as well as induction of anti-apoptotic protein Bcl-xL. These results suggest that in cerebral ischemia, C2dat1 stimulates the expression of CaMK2D and leads to an increase in CaMKIIδ protein expression. Over-expression of CaMKIIδ stimulates the IKKα/β-IκB-NF-κB pathway and transcriptional activation of Bcl-xL, leading to inhibition of ischemia-induced cell apoptosis^117^.

### FosDT

FosDT, also named MRAK159688, is a 604 nt lncRNA overlapping the downstream of gene Fos, which is located at chromosome 6 of rats^122^. FosDT expression in rats was highly upregulated during the acute period after focal ischemia in MCAO rats using arraystar lncRNA expression microarrays (about 13 folds of control). This upregulation was confirmed by Mehta et al. in 2015^123^. This increased expression of FosDT contributes to post-stroke brain damage and neurological dysfunction. Inhibition of FosDT resulted in the decrease in infarct volume and better post-ischemia motor function recovery compared with control group^123^, indicating that FosDT might be a cytotoxic factor for hypoxia injuries in rats. Bioinformatics analysis found that FosDT was congenic with *Fos* on chromosome 6q31 in rats^123^. *Fos* was found rapidly increasing after brain injury^124^. The increase in *Fos* was correlated with the increase in FosDT level, implying regulatory and/or transcriptional interactions between them. However, the detailed relations between FosDT and *Fos* still need further investigation.

Studies have shown that FosDT binds directly to chromatin-modifying proteins (CMPs) Sin3a and coREST (co-repressors of the transcription factor REST), two co-repressors for the transcription factor repressor element-1 silencing transcription factor (REST)^90^. REST is a repressor of neuronal traits such as neural differentiation and synaptic transmission^125^. In transient focal ischemia rats, REST formed a complex with coREST and Sin3a (REST-coREST-Sin3a) which inhibited the downstream genes of REST complex such as GluR2, NF-κB2, and N-methyl-D-aspartate 1 expression and increased ischemic brain damage^123^.

### SNHG14

LncRNA SNHG14, also named antisense of ubiquitin protein ligase E3A (UBE3A-ATS), is a 19.2 kb lncRNA located on chromosome 15q11.2. It is a host gene for two small nucleolar RNAs, C/D box 115 and 116 clusters. SNHG14 overlaps the entire UBE3A gene^126^. UBE3A is a brain-specific gene associated with neural development. The deficiency of UBE3A in children’s brain causes a neurogenetic disorder, Angelman syndrome^127^.

In neuronal differentiation, the decrease of UBE3A is associated with the over-expression of SNHG14^126^. In MCAO insulted mouse brain and OGD-treated Bv-2 microglia cell line, SNHG14 was significantly upregulated^128^. Accompanied by the increase in SNHG14, the expression of miR-145-5p decreased and phospholipase A2 group IVA (PLA2G4A) increased. Bioinformatics and in vitro experiments indicated that miR-145-5p binds directly to SNHG14 and PLA2G4A. PLA2G4A is a lipolytic enzyme belonging to the cytosolic phospholipase A2 (cPLA2) family. PLA2G4A plays a pro-inflammatory role in several diseases^129^ and is targeted by miR-145-5p. In MCAO mice and OGD-treated Bv-2 cells, lncRNA SNHG14 acted as an miRNA sponge. The increase in lncRNA SNHG14 sponged and inactivated the function of miR-145-5p, which resulted in a weaker suppression of miR-145-5p on PLA2G4A. The disinhibition of PLA2G4A then caused apoptosis and expression of pro-inflammatory factors such as tumor necrosis factor (TNF)-1α, nitric oxide (NO), and exacerbated neuron damage^128^.

### Gene ontology (GO) term enrichment analysis for potential functions of these nine lncRNAs

GO term enrichment is a widely used bioinformatic tool for interpreting sets of genes to a set of predefined terms in order to better understand the underlying biological processes of some genes. Even though part of the roles of nine observed lncRNAs has been discovered, more potential functions are still unknown. We performed a GO enrichment analysis to predict more functions and mechanisms of these nine lncRNAs. As shown in Table 2, four of the nine lncRNAs were enriched in different GO terms. Among these four lncRNAs, MEG3 has the most predicted functions. MEG3 was predicted to be related to biological processes including organ development (lung, liver, embryo, skeletal muscle development), vascular functions (angiogenesis and VEGF pathway), gene regulation (DNA transcription, RNA folding, methylation, and gene imprinting), inflammation, and cell growth. It is well understood that prevention of ischemic injury and promotion of neurogenesis are two main strategies for ischemic stroke management. According to the GO enrichment results, MEG3 may also interfere with neuroregeneration, angiogenesis, and inflammation through mechanisms of gene regulation (transcription, RNA folding, and methylation). H19 is another lncRNA with a predicted role in neuroregeneration through regulation of cell proliferation and gene expression. MALAT1 was predicted to be related to synapse organization.

**Table 2:** GO enrichment analysis of some ischemic stroke-related lncRNAs

**LncRNA**	**GO category**	**GO term**
MEG3	Biological process	**DEVELOPMENT:** lung development; embryo development; post-embryonic development; skeletal muscle development; liver development; multicellular organism growth; determination of adult lifespan.
		**VASCULAR:** angiogenesis; regulation of vascular endothelial growth factor receptor signaling pathway;
		**GENE REGULATION:** RNA folding; DNA-templated transcription; DNA biosynthesis; hypomethylation of CpG island; DNA methylation; genetic imprinting.
		**INFLAMMATION:** Notch signaling pathway; SMAD protein signal transduction.
		**CELL GROWTH:** cell differentiation; negative regulation of cell proliferation.
		**OTHERS:** axon guidance; gonadotropin secretion; epithelial tube morphogenesis.
	Molecular function	Transcription co-activator activity.
	Cellular component	Nucleus, cytoplasm.
MALAT1	Biological process	Nuclear speck organization; mRNA splicing; synapse organization; myoblast proliferation.
	Molecular function	Protein binding.
	Cellular component	Nuclear body, nuclear speck.
H19	Biological process	**GENE REGULATION:** DNA methylation; gene expression; genetic imprinting.
		**CELL GROWTH:** regulation of multicellular organism growth; regulation of cell proliferation.
	Cellular component	Polysome, cytoplasm.
TUG1	Biological process	Photoreceptor cell development.

## Conclusion and perspectives

Taken together, lncRNAs play important roles in ischemic stroke by modulating cell survival, inflammation process, and angiogenesis (Fig. 3). Our new GO enrichment analysis provides a new direction on the functions and mechanisms of these lncRNAs in ischemic stroke.

In recent years, great progress has been made to uncover the potential roles of lncRNAs in ischemic stroke. Besides the nine well-studied lncRNAs, a bulk of aberrantly expressed lncRNAs were identified using new technologies such as RNA-seq, deep sequencing, and microarray. Two-hundred and ninety-nine lncRNAs were found differentially expressed in the whole blood of ischemic stroke patients using microarray^47^; hundreds of aberrantly expressed lncRNAs were found in the brain of ischemic insulted animal models and in OGD cell models^48,130,131^. These big data sets showing lncRNA changes in ischemic stroke are existing, and provide a unique view of the pathobiology of ischemic stroke. Elucidating the functions and mechanisms of these lncRNAs in biological systems under normal and pathological conditions may lead to potential opportunities for identifying biomarkers and novel therapeutic targets of ischemic stroke.

However, this field is still facing a lot of challenges. Till now, only a very small number of lncRNAs have been studied for their effects in the pathological process of ischemic stroke. Large-scale loss-of-function and gain-of-function studies are needed to demonstrate lncRNA functions. In addition, there is a lack of comprehensive and reliable public lncRNA databases of all known lncRNAs in humans and commonly used model organisms; thus, it remains difficult to cross reference lncRNAs from several disparate databases (such as RefSeq and Ensembl) and from primary publications. Studies in lncRNAs require more sensitive methods of detections as compared to protein and other RNAs due to their lower expression. Application of the new tools like CRISPR-Display and bioinformatics advances facilitates the function research of lncRNAs^132^. It is anticipated that the field of lncRNA research continues to improve in near future.

Stroke is one of the leading causes of death and adult disability. Screening for lncRNAs and elucidating the functions and mechanisms of lncRNAs in ischemic stroke may provide a better understanding of its cellular and molecular pathophysiological process. LncRNAs may act as biomarkers, therapeutic target, or a novel epigenetic intervention tool for the treatment and prevention of ischemic stroke.
